# The NLR member CIITA: Master controller of adaptive and intrinsic immunity and unexpected tool in cancer immunotherapy

**DOI:** 10.1016/j.bj.2023.100631

**Published:** 2023-07-17

**Authors:** Greta Forlani, Mariam Shallak, Andrea Gatta, Amruth K.B. Shaik, Roberto S. Accolla

**Affiliations:** Laboratories of General Pathology and Immunology “Giovanna Tosi”, Department of Medicine and Technological Innovation, School of Medicine, University of Insubria, 21100 Varese, Italy

**Keywords:** CIITA, NLR, Restriction factors, HTLV-1, Cancer

## Abstract

Human nucleotide-binding oligomerization domain (NOD)-like receptors (NLR) include a large family of proteins that have important functions in basic physio-pathological processes like inflammation, cell death and regulation of transcription of key molecules for the homeostasis of the immune system. They are all characterized by a common backbone structure (the STAND ATPase module consisting in a nucleotide-binding domain (NBD), an helical domain 1 (HD1) and a winged helix domain (WHD), used by both prokaryotes and eukaryotes as defense mechanism. In this review, we will focus on the MHC class II transactivator (CIITA), the master regulator of MHC class II (MHC-II) gene expression and the founding member of NLR. Although a consistent part of the described NLR family components is often recalled as innate or intrinsic immune sensors, CIITA in fact occupies a special place as a unique example of regulator of both intrinsic and adaptive immunity. The description of the discovery of CIITA and the genetic and molecular characterization of its expression will be followed by the most recent studies that have unveiled this dual role of CIITA, key molecule in intrinsic immunity as restriction factor for human retroviruses and precious tool to induce the expression of MHC-II molecules in cancer cells, rendering them potent surrogate antigen presenting cells (APC) for their own tumor antigens.

## Highlights

CIITA is a peculiar NLR member.
• CIITA acts as master controller of adaptive immunity.
• CIITA acts as crucial molecule of intrinsic immunity as restriction factor against human retroviruses.

## Introduction

### CIITA discovery, structure and function

Human nucleotide-binding oligomerization domain (NOD)-like receptors (NLR) constitute a rather heterogenous family of proteins that have evolved both in eukaryotes and prokaryotes as basic elements of defense against aggressors [[1], [2], [3], [4]]. Most of them serve function related to innate immunity with the distinguished exception of the MHC-II and the MHC-I transactivators which are instead regulators of adaptive immunity [[5], [6], [7]]. In this review, we will focus on the MHC-II transactivator (CIITA). CIITA is included in the NLR transcription factor family [5,7] as the unique member of the NLRA subfamily [3]. The discovery of CIITA, its function as dominant regulator of MHC-II gene expression, the chromosomal location of the locus encoding it, designated Air1, was done in our lab between 1983 and 1986. We took advantage of the isolation of a B cell mutant from the B cell line Raji, designated RJ2.2.5, that had lost expression of all MHC-II genes after irradiation and immunoselection [8]. After complementation with normal mouse B cells by somatic cell fusion or transfection of high molecular weight DNA from human B cells, we could show the re-expression of all human MHC-II genes in RJ2.2.5 cells [[9], [10], [11]]. The cloning of the mRNA encoding CIITA was performed by Steimle et al. in 1993 using RJ2.2.5 as acceptor of a cDNA library [12].

CIITA has a tripartite domain structure similar to that of other NLR proteins with an N-terminal caspase-like activation and recruiting domain (CARD)-like and an acidic activation domain (AD), a core signal transduction ATPase with numerous domains (STAND) and a leucin rich domain (LRR) [3]. The CARD domain is present in the longest form of CIITA gene that is specific for dendritic cells (see below). In general CARD domains allow interaction with proteins containing similar CARD domains. The leucin rich repeat (LRR) found in all NLR family members is thought to be important in heterologous protein–protein interactions which is a distinctive characteristic of this family. The nucleotide binding domain (NBD) which is a component of the more structured STAND module drives the self-oligomerization of the protein and is important for cellular defense across species from prokaryotes to eukaryotes [[3], [4], [5]] CIITA contains both nuclear localization signals (NLS) and nuclear export signals (NES). Indeed, unlike other NLR proteins that are mostly localized in the cytoplasm, or associated with mitochondria or the plasma membrane, CIITA can shuttle between nucleus and cytoplasm [13].

**Fig. 1:**
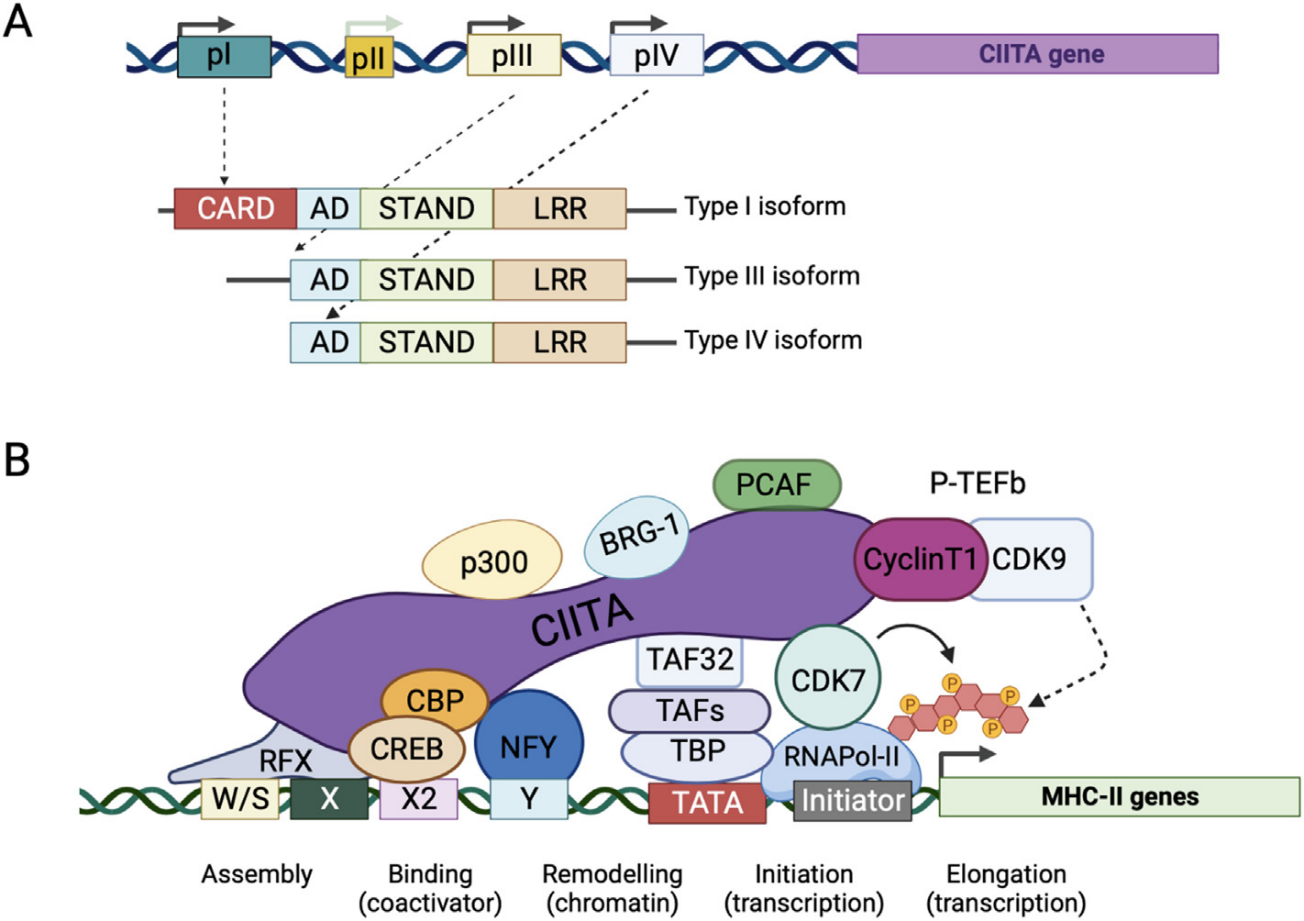
**The MHC class II transactivator CIITA: schematic structure of the gene and function of the protein.** (A) Expression of the CIITA gene is controlled by three independent promoters (pI, pIII and pIV) having different functions and expressed in specific cell types. pII is not associated to any transcript. The three types of mRNA encoding CIITA are derived from pI, pIII and pIV, and they encode three different protein isoforms (Type I, Type II and Type III), which differ only at their N-terminal ends. The region shared by all three isoforms contains an acidic activation domain (AD), a core signal transduction ATPase with numerous domains (STAND) and a leucin rich domain (LRR). The N-terminal region of the Type I isoform encodes an additional caspase-like activation and recruiting domain (CARD), not present in the Type II and III protein isoforms. (b) CIITA leads to the assembly of the MHC-II enhanceosome by interacting with CREB/CBP and the NF-Y and RFX transcription factors associated with W/SXY module. In addition, CIITA binds to chromatin modifying enzymes, as BRG1 and many transcriptional co-activators including histone deacetylases (HDACs), histone methyltransferases (HMTs) and histone acetyltransferases (HATs), as p300 and PCAF, thus contributing to chromatin remodeling and transcription activation. Furthermore, CIITA regulates the initiation of MHC-II transcription by recruiting several components of the canonical TFIID complex, as TAFs proteins and TBP. By interacting with Cyclin T1 and CDK9 of positive transcription elongation factor-b (P-TEFb) complex and CDK7, CIITA also controls the elongation of primary transcripts. Figure created with BioRender.com.

CIITA expression is under the control of several promoters that show cell type specificity [Fig. 1A] [14]. Lack or defective expression of CIITA leads to a rare but extremely severe form of immunodeficiency, the Bare Lymphocyte syndrome (BLS), characterized by impaired adaptive immune response during the first years of life and often incompatible with life [15]. CIITA is not a DNA-binding protein but exerts its principal biological function by coordinating the binding of transcription factors on the MHC-II gene promoter [Fig. 1B] [16]. It interacts with a multi-protein complex which includes the regulatory factor complex X (RFX-5, RFX-ANK and RFX-AP), the cAMP-responsive-element-binding protein 1 (CREB1), the activating transcription factor 1 (ATF1) and nuclear transcription factor Y complex (NF-YA, NF-YB, NF-YC). All these factors bind to a conserved DNA sequence module, designated W/SXY, located in the upstream regions of all MHC-II genes, to form the MHC-II enhanceosome [17,18]. In addition, CIITA binds to, and acts as a platform for the recruitment of many transcriptional co-activators including histone acetyltransferases (HATs), histone deacetylases (HDACs), histone methyltransferases (HMTs), chromatin remodeling factors as well as factors of the general transcription complex [19], which modulate the activity of enhanceosome and alter chromatin accessibility, further regulating the transcription of MHC-II genes [20,21]. Furthermore, the interaction of CIITA with the positive transcription elongation factor-b (P-TEFb), composed of Cyclin T1 and CDK9, endows the MHC class II transactivator with a role also in the elongation of primary transcripts [22].

CIITA acts as a master regulator of not only polymorphic MHC-II genes but also accessory genes including invariant chain, HLA-DM and HLA-DO, involved in antigen processing and presentation [23,24]. CIITA controls both the constitutive and the inducible expression of MHC-II genes. The constitutive MHC-II expression is tightly regulated and limited to specific cell types, including thymic epithelial cells and professional antigen presenting cells (APC) such as dendritic cells (DC) and B lymphocytes. The inducible MHC-II expression can be seen in activated T lymphocytes by a variety of mitogenic stimuli, in macrophages, another class of APC, and non-haemopoietic cells by treatment with interferon gamma (IFN-γ). Both constitutive and inducible MHC-II expression are indeed dependent on the constitutive and inducible expression of CIITA, respectively.

Regulation of CIITA gene expression has been extensively studied and CIITA promoter knock-out mice have significantly contributed to unveil the complexity of CIITA gene transcription. CIITA gene expression is regulated by four independent promoters, named promoter I, II, III and IV, that can be individually activated in a cell type- and stimulus-specific manner. Three of these promoters are highly conserved between the human and mouse gene (pI, pIII and pIV). In contrast, pII has only been found in the human gene where it has been shown to display only very low transcriptional activity. The different promoters do not share sequence homology and are not co-regulated [25]. Each of these promoters controls the transcription of a unique first exon, resulting in the production of three different CIITA transcripts that differ at their amino terminus [Fig. 1A], indicating that CIITA activity is regulated in a complex fashion by both cell-specific promoters as well as cell-specific isoforms. The shared second exon contains a translation initiation codon that can be used in all three types of transcripts to generate a 1106 amino acid protein with an apparent molecular weight of 121 kDa. However, an additional in frame translation codon is present in the first exons of the type I and type III transcripts and gives rise to two additional protein isoforms of 1207 and 1130 amino acids, respectively [25]. The usage of the different CIITA promoters leads to the constitutive and the inducible expression of MHC-II genes in professional APCs and in non-professional APC, respectively. CIITA type I mRNA, resulting from the use of promoter I, is highly expressed in cells of myeloid origin as conventional DC and IFN-γ-activated macrophages, and leads predominantly to the expression of the longest isoform of 132 kDa. During DC maturation the transcriptional activity of pI is significantly repressed as a consequence of histone deacetylation of a large domain encompassing the entire regulatory region of the gene [26]. The N-terminal extension of 101 aa found in type I isoform encodes a caspase-like activation and recruiting domain (CARD), which seems to be responsible to the more efficient capacity of type I isoform in activating MHC-II promoters [27]. This CARD-like domain reinforces the similarity between CIITA and other NLR proteins, as many of which contain an N-terminal CARD [3]. The 124 kDa B cell CIITA isoform is transcribed by promoter III, which is also used by activated T cells in humans and plasmacytoid DC. Within pIII, two functional proximal promoter elements named Activation Responsive Element (ARE)-1 and ARE-2 are sufficient to drive the expression of CIITA in B cells [28]. However, an extended region of 5 kb upstream to pIII is required for IFN-γ-dependent CIITA expression in macrophage/monocytic lines in a STAT1-dependent fashion, and for the constitutive and IFN-γ-mediated MHC-II expression in melanomas and glioblastomas [[29], [30], [31]]. Interestingly, pIII induction by IFN-γ is species-specific, since it is not operative in mice, probably because the IFN-γ-responsive enhancer that is found upstream of human pIII is not present in the mouse gene [32]. Moreover, pIII is transcriptionally active in human activated T cells but not in mouse T cells [33,34]. In non-hematopoietic cells, the expression of CIITA can be mainly induced by IFN-γ through the usage of pIV, on which three transcription factors, the signal transducer and activator of transcription 1 (STAT-1), IFN-regulatory factor 1 (IRF-1) and upstream transcription factor 1 (USF-1) are recruited. These transcription factors bind to specific regulatory elements, located on conserved proximal region of the promoter. The IFN-γ-activated site (GAS) and the E box are bound cooperatively by STAT1 and USF-1, while the IFN-regulatory factor (IRF) element (IRF-E) is co-occupied by IRF1 and IRF2 [29]. This IFNγ-induced expression of CIITA can be downregulated by several cytokines, including transforming growth factor-β (TGFβ) and interleukin-10 (IL-10) [35]. In B cells, a further level of regulation of CIITA expression is represented by 5′ untranslated (5′UTR) region of CIITA-pIII, which contains three c-AMP-responsive elements (CRE), bound by the activating transcription factor (ATF)/cAMP-responsive element (CRE) binding protein (CREB). Deletion of the 5′-UTR resulted in severe reduction of constitutive transcriptional activation of type III CIITA isoform in Raji and Ramos B cells, but not in Jurkat T cells [36]. Developmentally, CIITA is silenced when B cells differentiate into plasma cells [37,38]. In addition to ARE1, ARE2 three other cis-regulatory elements, named site A, B, and C, located between −545 bp and +113 bp of the pIII transcription initiation site are essential for B cell-specific CIITA expression. These elements are bound by E-box-binding factor (E47), PU.1/IRF1/IRF4, SP1, CREB/CBP, and Oct1. Following differentiation to plasma cells, the repressor factor Blimp-1 binds to this region and displaces the above activator proteins, causing suppression of CIITA transcription [39,40]. In addition to the important role of the CIITA 5′ regulatory regions, CIITA expression in myelomonocytic cells, but not in B cells, is regulated by a cis-acting region of transcript instability mapped within the distal 650 bp of the CIITA 3′UTR mRNA. Phorbol ester treatment strongly promotes CIITA mRNA degradation in both U937 and THP-1, two distinct myelomonocytic cell lines, whereas it does not affect at all the CIITA mRNA stability of Raji B cells [41]. Epigenetic regulation of CIITA is also an important element in the expression of this protein and has a major impact on MHC-II expression in tumor cells possibly facilitating immune escape. Indeed, epigenetic control by DNA methylation of promoter III and promoter IV in T cell malignancies is often found. Interestingly, developmental tumor cell lines such as teratocarcinomas and neuroblastomas as well as some differentiated glioblastomas, carcinomas and melanomas cell lines often display an MHC class II negative phenotype, even after treatment with IFN-gamma. Molecular analysis has shown that this was due mostly to hypermethylation of CIITA promoter IV [42]. Furthermore, in squamous-cell carcinoma cell lines histone deacetylation may also contribute to CIITA silencing [43].

Epigenetic mechanisms of CIITA silencing, particularly in tumor cells, may represent a way to avoid tumor antigen recognition by the immune system and thus elusion from anti-tumor immune response.

Although CIITA is the major regulator of MHC class II gene expression other factors may influence the expression of these genes and their products. First, at transcriptional level, mutation in component of the RFX complex result in lack of expression of MHC class II genes [14]. Second, defects in expression of the invariant chain result in altered or even abolished transport of the MHC class II heterodimers to the specific intracellular compartments where MHC class II molecules encounter potential binding antigenic peptides. This results in altered cell surface expression of MHC class II molecules [44]. Third, even the amount of the already cell surface expressed MHC class II molecules can be regulated. For example, in human monocytes IL-10 increases the expression of a ubiquitin ligase membrane-associated RING-CH1 (MARCH-1) that ubiquitinates the tail of cell surface MHC II molecules initiating their rapid internalization and destruction [45].

## CIITA as restriction factor

During evolution, immunity against pathogens has progressed through a series of continuous adaptations of both the host versus the pathogen to counteract its virulence and of the pathogen versus the host to elude its mechanisms of defense. In most cases, particularly during virus infection, these mechanisms of mutual adaptation have allowed persistence of both species because prevalence of one versus the other would have had as result elimination of either the host or the pathogen [46]. As far as the host defenses, two basic mechanisms of immunity, innate and adaptive, have been extensively described. Basically, this distinction relies on the assumption that innate immunity acts in a rather non-specific way as opposed to adaptive immunity that utilizes effector mechanisms, cellular and humoral, specific for the pathogen [47,48]. Nevertheless, this distinction is rather artificial as cells and molecules of innate and adaptive immunity often cooperate each-other and often trigger each-other, again showing a concerted evolution for the protection of the host [49].

**Fig. 2:**
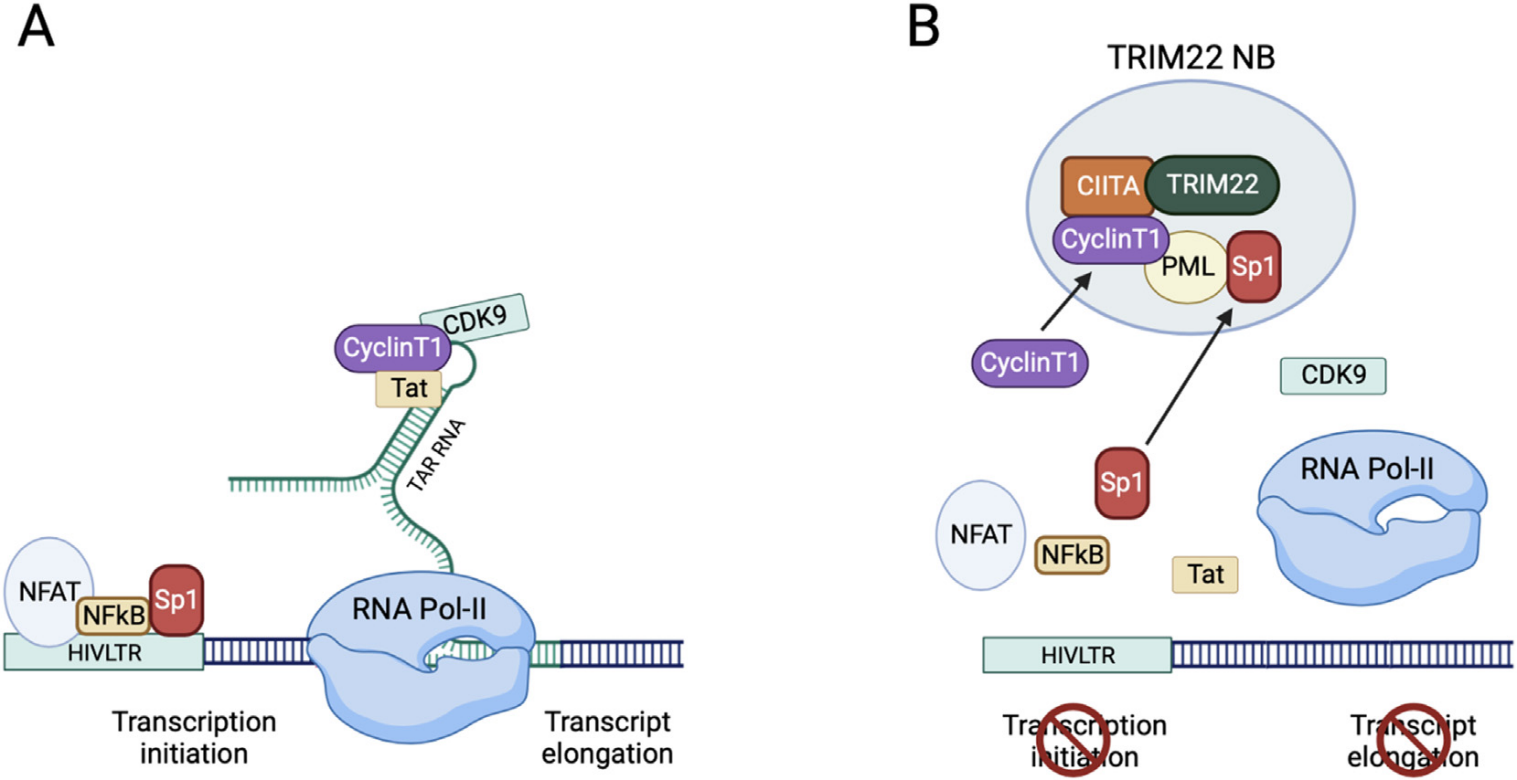
**CIITA is a restriction factor for human retroviruses: action on HIV.** (A) The transcription of HIV-1 depends on a complex interplay between numerous viral and cellular factors. Cellular transcription factors such as nuclear factor kappa B (NF-κB), nuclear factor of activated T cells (NFAT), and specificity protein 1 (Sp1) bind to viral LTR and are crucial to initiate viral genome transcription. The viral transactivator Tat interacts with the Trans-Activation Response (TAR) region at the 5′ end of the nascent viral RNA and recruits the P-TEFb complex made by Cyclin T1 and CDK9 and RNA polymerase II (RNA Pol-II) to promote elongation of the primary transcript (B) CIITA binds to Cyclin T1 and displaces it from the P-TEFb complex. CIITA also binds another restriction factor, TRIM22, which drives the complex to specific nuclear bodies (TRIM22 NB) where CyclinT1 is also bound and further retained by promyelocytic protein PML. TRIM22 NB represent also a docking place for Sp1 whose detachment from the viral LTR due to interference mediated by TRIM22 contributes to drastically reduce basal HIV-1 transcription. The convergence of several restriction factors, particularly CIITA and TRIM22, acting in synergy to displace both basal transcription and transcript elongation factors in a single nuclear body represents an unprecedented finding in intrinsic immunity. Figure created with BioRender.com.

A third form of immunity, designed intrinsic immunity, acts in parallel to the two major forms of protection, is mostly focused against viruses and relies on intracellular molecules either constitutively expressed or induced by mediators of innate immunity, and defined as restriction factors (RFs). The function of RFs is to counteract distinct steps of virus life cycle [50,51]. Although a consistent part of the described NLR family components act either as innate or intrinsic immune sensors, CIITA stands alone for its function. The originality, peculiarity and unicity of CIITA resides in its dual function not only as regulator of adaptive immunity but also as restriction factor for human retroviruses and thus as a master factor of intrinsic immunity. Whether the first function preceded or followed the second function during evolution is still unexplored. The first suggestion that CIITA was acting as a RF for human retroviruses came from our original observation that CIITA inhibits HIV-1 replication in human T cells by competing with Cyclin T1 of the P-TEFb complex, a crucial host factor recruited by the viral transactivator Tat to elongate transcripts [52]. More recently we were able to show that CIITA was inhibiting viral replication also in human macrophages, another important cellular target of HIV-1 infection [53]. These experiments unveiled an unprecedented feature of retrovirus restriction: the concerted action of several restriction factors including CIITA, TRIM22 and PML in counteracting HIV-1 replication by binding each other and segregating Cyclin T1 in a previously undescribed intranuclear body, thus preventing, in a synergistic way, elongation of viral transcripts and HIV-1 replication [Fig. 2] [54,55].

**Fig. 3:**
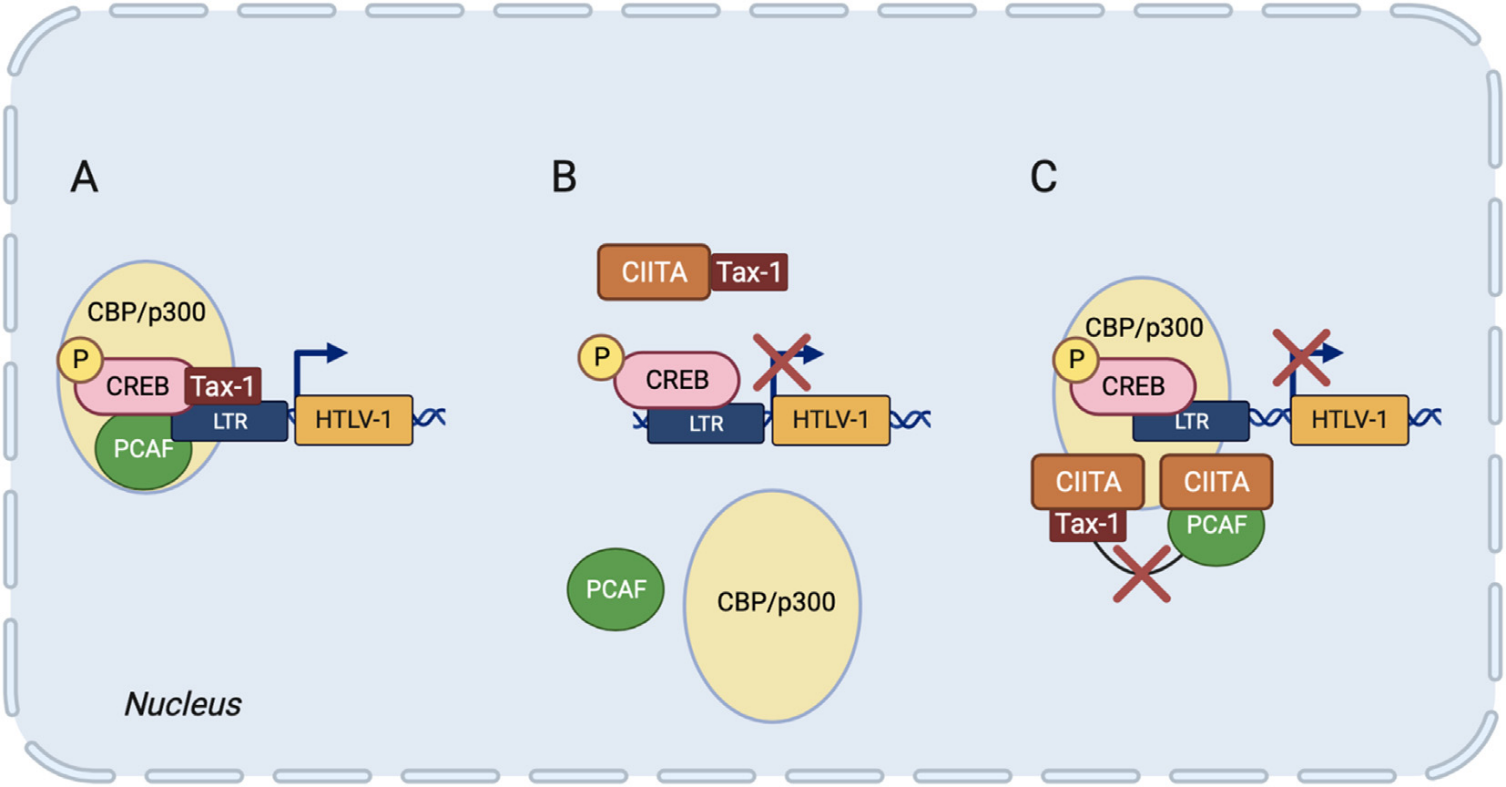
**CIITA is a restriction factor for human retroviruses: action on HTLV-1.** Models of inhibition of Tax-1-mediated HTLV-1 transcription by CIITA. (A) Tax-1 activates proviral transcription by recruiting several cellular factors such as CREB, CBP, p300 and PCAF on HTLV-1 LTR promoter. (B) CIITA inhibits Tax-1-mediated HTLV-1 transcription by physically interacting with Tax-1 and preventing its association with CREB, CBP, p300 and PCAF and their recruitment on viral promoter. (C) In a non-mutually exclusive model, the interaction between CIITA and Tax-1 does not prevent the recruitment of the viral transactivator on viral LTR. However, in this case Tax-1 is not efficient at promoting HTLV-1 transcription because its association with PCAF is weak due to the steric hinderance caused by the Tax-1-CIITA and/or CIITA/PCAF interactions. Figure created with BioRender.com.

Similarly to HIV-1, CIITA acts as restriction factor for the human T cell leukemia virus HTLV-1, the first discovered human retrovirus responsible of a still untreatable form of blood cancer designated Adult T cell Leukemia/Lymphoma (ATL) [56], and for the closely related HTLV-2 [57]. In these cases, the molecular mechanisms of restriction are similar but not identical to the restriction operated on HIV-1. Similarly to HIV-1, the viral transactivators Tax-1 from HTLV-1 and Tax-2 from HTLV-2 are the major targets of the CIITA-mediated restriction. While HTLV-2 replication is inhibited by CIITA toward the formation of a molecular complex between CIITA and the nuclear factor NF-YB that binds Tax-2 and prevent the Tax-2-mediated LTR transactivation [58], a more diversified action of CIITA is exerted on HTLV-1 Tax-1. Tax-1 interacts with several cellular factors, involved in various pathways of activation and/or repression of transcription [Fig. 3A] [59]. Importantly, most of these factors, such as the histone acetyl transferases p300, CBP, and PCAF, are also used by CIITA to promote MHC class II gene transcription [59]. It was found that overexpression of PCAF, but not of p300, counteract the CIITA inhibitory action on Tax-1, restoring transactivating function of the viral protein. Most importantly, CIITA directly interacts with Tax-1, thus greatly contributing to the displacement of the viral transactivator from the viral promoter and consequently to the assembly of host factors necessary to initiate and stabilize the transcription of the viral genome [Fig. 3B,C] [60]. In line with the idea that CIITA interferes with the recruitment of critical host transcription factors on viral promoter, it was shown that overexpression of CREB and ATF1, both required for the assembly of the functional complex necessary for Tax-1-mediated activation of HTLV-1 LTR promoter, counteracted the inhibitory action of CIITA on Tax-1 [60]. These findings corroborated the idea that CIITA acts as HTLV-1 restriction factor by counteracting the physical and functional interaction between Tax-1 and key factors needed to promote Tax-1-mediated HTLV-1 LTR transactivation.

**Fig. 4:**
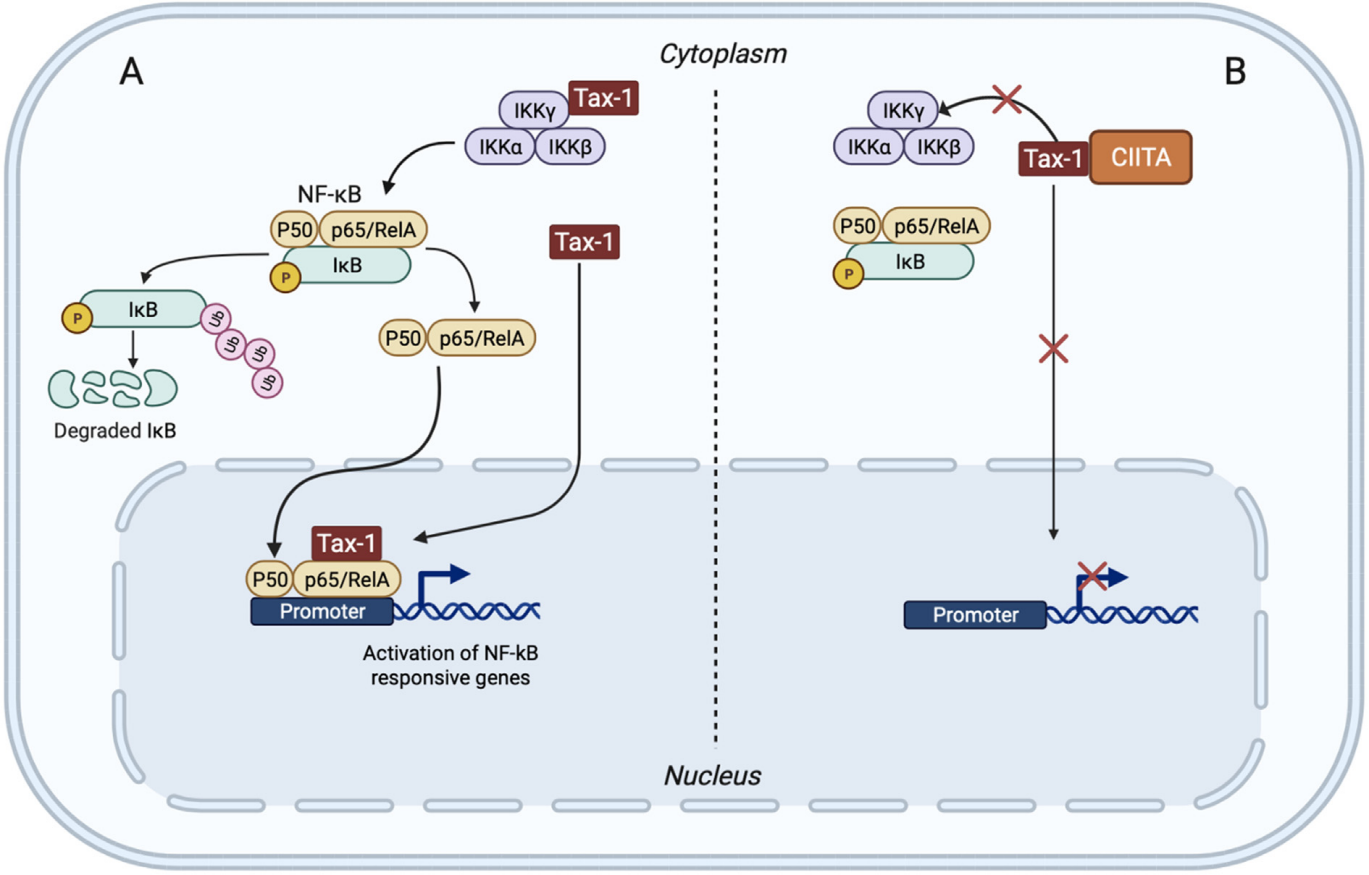
**CIITA may counteract the oncogenic properties of HTLV-1.** The oncogenic potential of Tax-1 is mainly correlated to its ability to activate the NF-kB pathway, by acting both at cytoplasmic and nuclear level. In the cytoplasm Tax-1 binds to IKKγ subunit of the trimeric IKK complex, activating the kinase activity of IKKα and IKKβ subunits. The activation of IKK complex results in phosphorylation and proteasomal degradation of the cytoplasmic inhibitor (IκB) of NF-kB and the translocation of the NF-κB dimers (p50-p65/Rel (A) to the nucleus where it activates the transcription of NF-kB responsive genes. In the nucleus, Tax-1 binds to p65/RelA and stabilizes its association to the NF-kB responsive promoter, further promoting gene transcription. (B) By interacting with Tax-1, CIITA suppresses Tax-1 oncogenicity both at cytoplasmic and nuclear levels by (a) preventing its association and the subsequent activation of IKK complex, (b) impairing its nuclear translocation and thus its interaction with p65/RelA. Figure created with BioRender.com.

The finding that CIITA directly interacts with Tax-1 was the reason to investigate the other crucial aspect of HTLV-1-host interaction, that is the involvement of HTLV-1 in the oncogenic process leading to ATL [56,61]. It is believed that a consistent part of the Tax-1-mediated oncogenicity is due to the constitutive activation of the NF-kB pathway by the viral transactivator [Fig. 4] [62]. Since CIITA binds to and inhibits the action of Tax-1 it was important to assess the role of CIITA on the Tax-1 mediated activation of NF-kB. It was found that endogenous CIITA inhibits the activation of the canonical NF-kB pathway by Tax-1 in various ways and both in the cytoplasm and the nucleus [63]. First, CIITA retains a large proportion of Tax-1 in the cytoplasm with consequent impaired migration of the NF-kB component RelA into the nucleus. Second, nuclear CIITA associates with Tax-1/RelA in nuclear bodies, blocking Tax-1-dependent activation of NF-kB-responsive genes [Fig. 4]. These results reinforce the notion of CIITA as a polyvalent restriction factor which in the case of HTLV-1 may also counteract Tax-1-mediated oncogenicity [63,64]. The intrinsic inhibitory activity of CIITA is not limited to human retroviruses. A strong association between CIITA and inhibition of viral infection and spreading was also recently observed for Ebola virus and Coronavirus infection. In this case the MHC class II transactivator does not function as a restriction factor but as an activator of transcription of the invariant chain p41 subunit that in turn induces viral resistance. The mechanism of inhibition involved a direct action of p41 in the endosome on the activity of cathepsins [65], as these enzymes are needed to process the Ebola glycoprotein as well as the S protein of SARS-CoV-2 for an efficient viral fusion.

## CIITA as a tool to increase tumor immunogenicity

**Fig. 5:**
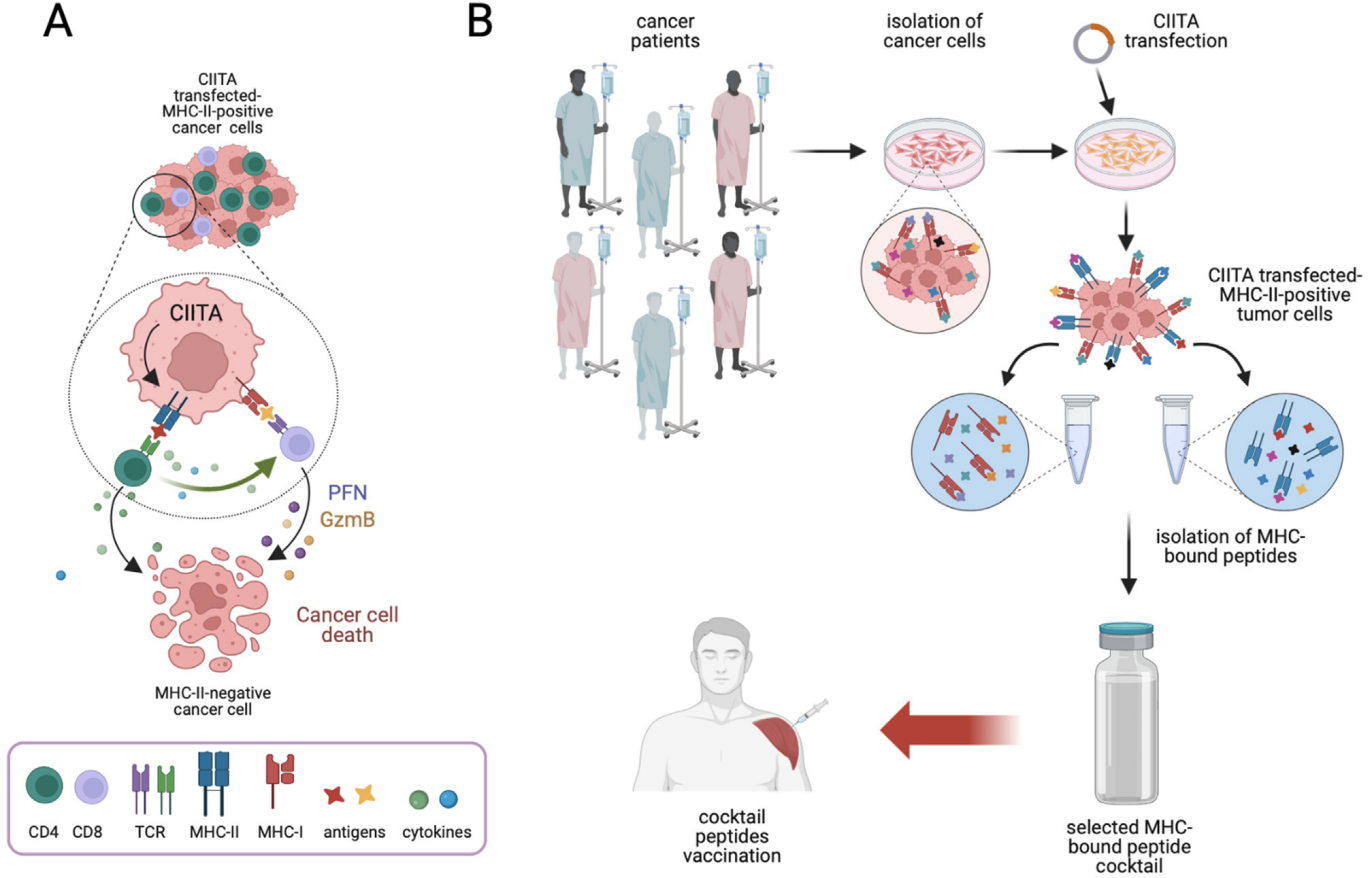
**CIITA and cancer.** A) CIITA-modified cancer cells express functional MHC class II molecules and can function as surrogate antigen presenting to trigger and activate tumor specific CD4+ T helper cells, basic mediators of adaptive immunity. Downstream secretion of cytokines and particularly triggering of cytolytic T cells (CTL) leads to elimination of tumor cells. B) CIITA-modified cancer cells represent an unprecedented source of MHC-II bound tumor peptides that may be used for the formulation of novel anti-tumor vaccines. Tumor cells isolated from cancer patient are MHC-II-negative and express low levels of MHC-I molecules on cell surface. Tumor peptides potentially boundable to MHC-II molecules are hidden into cancer cells. Upon CIITA transfection, cancer cells express MHC-II molecules that can now bind hidden tumor specific peptides and present them on the cell surface. Both MHC-II and MHC-I bound peptides can be eluted, purified and selected on the basis of their exclusive expression in tumor but not in normal cells. After immunogenic validation, a selected group of tumor-specific peptides can be used to generate a vaccine cocktail to be tested in clinical setting as therapeutic vaccine in cancer patients. Perforin (PFN). Granzymes (GzmB). Figure created with BioRender.com.

As regulator and coordinator of MHC class II gene expression, CIITA is a master controller of antigen presentation and thus a crucial factor in adaptive immunity. As described above, in physiological conditions, CIITA is expressed constitutively in B cells and dendritic cells and in inducible fashion in macrophages. These cell types, the professional antigen presenting cells (APC), are capable to trigger the response of the key cell in adaptive immunity, the antigen-specific MHC-II-restricted CD4+ T helper cell (Th). Without triggering and activation of Th cells, none of the effector function of adaptive immunity, that is antibody production by B cells and the MHC class I-restricted cytolytic function of CD8+ T cells (CTL), can be initiated and maintained. For many years it was believed that the capacity to process and present antigenic peptides to Th cells was exclusively focused on the engulphed exogenous antigenic proteins by the APC and in this role DC and, partially, macrophages were the key cells capable to prime naïve antigen specific Th cells [66]. Presentation of endogenous peptides, such as those derived by altered proteins synthesized by the cell, as tumor antigens are, and non-self viral proteins synthesized by the integrated genome of viruses was instead believed to be exclusively mediated by MHC class I molecules, expressed virtually in all cells and presented to CTL. Many exceptions to the above rules, both for MHC class II and MHC class I presentation pathways were however described with exogenously derived peptides presented by MHC class I and endogenously derived peptides presented by MHC class II [[67], [68], [69]]. The capacity of MHC-II molecules to present endogenous peptides lead us to conceive a rather unorthodox approach to induce and/or increase the capacity of tumor cells to be “seen” by tumor specific Th cells and thus trigger a possibly more efficient anti-tumor immune response. Most tumor cells, both carcinoma and sarcomas, do not express MHC-II molecules because of lack of expression of CIITA. The idea was to modify tumor cells by transducing them with of CIITA and thus render them MHC-II-positive, closely mimicking the physiological expression of MHC-II molecules. In this we were motivated by early results showing the human tumor cell lines expressing MHC-II molecules after transfection with CIITA could process and present peptides from *M. Tuberculosis* Ag85 protein to antigen-specific CD4+ T cell clones [70]. In a large series of studies in animal experimental models we were able to show that genetic transfer of CIITA into tumor cells of distinct histotype and different H-2 genetic background not only could invariably induce MHC-II gene expression but also could elicit tumor rejection or strong retardation of tumor growth when CIITA-expressing tumor cells were injected into syngeneic immunocompetent hosts [Fig. 5A] [71,72]. Recently, this was verified also in one of the most malignant tumor, the glioblastoma, that has the additional complexity of developing into the brain, an organ considered for long time protected from immune attack by the blood brain barrier [73]. In deep analysis of the mechanisms leading to tumor rejection demonstrated crucial aspects of the immune response induced by CIITA-expressing tumor cells. First, immunity to CIITA-tumor cells was long lasting and capable to counteract the growth of parental CIITA-negative tumor cells [[71], [72], [73]]. Second, the tumor microenvironment generated by injection of CIITA-tumor cells was dramatically different from the one generated by parental cells in that a much stronger infiltration of lymphocytes was observed, with rapid infiltration of CD4+ Th cells preceding infiltration of DC and CD8+ T cells [74]. Third, and more importantly, anti-tumor immunity could be transferred to syngeneic naïve recipients by adoptive cell transfer of immune cells and particularly by CD4+ Th cells [72,75]. All these findings strongly suggested that CIITA-induced MHC-II expression in tumor cells made these cells surrogate APC of their own tumor antigens *in vivo.* The final proof came from experiments in which dendritic cells and macrophages could be functionally deleted *in vivo* in C57/BL6 mice to eliminate the crucial APC that prime antigen specific naïve Th cell. In fact, these animals injected with CIITA-tumors could still reject and/or strongly retard the growth of tumor cells [76]. These results have profound immunological implications both from a theoretical and practical point of view. They establish that macrophages, B cells and particularly DC, are not the exclusive antigen processing and presenting cells that prime naïve Th cells for the initiation of the adaptive immune responses [66]. Other cells, in this case tumor cells, can efficiently process and present endogenous peptide antigens to naïve Th cells provided they express MHC-II molecules in a “physiological” fashion, that is under the control of CIITA [77]. They also demonstrate that immunogenic tumor-specific peptides hidden within the tumor cell, can be bound by MHC-II molecules and forced to be exposed to the cell surface for scrutiny by Th cells [78,79]. This opens the way to use CIITA-tumor cells as source MHC-II-bound tumor peptides for the preparation of newly conceived anti-tumor vaccines to optimize the response against the tumor in clinical settings [Fig. 5B] [80].

## Conclusions

The discovery of MHC-II transactivator CIITA has marked a crucial advance in the understanding of the basic mechanisms controlling adaptive immunity. Its role on the expression of MHC-II genes has disclosed the complex mechanism through which CIITA acts as coordinator and integrator of expression at several levels including the assembly and binding of transcription factors to the MHC-II promoter, the remodeling of chromatin as well as the facilitation of the initiation of transcription and the elongation of primary transcripts. The fine regulation of its expression has helped to identify the existence of several promoters involved in its cell-specific and developmental-specific expression so crucial for the various functions of modulation of adaptive immunity. Its most recently discovered function as restriction factor of retrovirus replication and spreading has allowed to identify its unprecedented role as mediator of intrinsic immunity and thus as a rather unique player within the family of NLR. May be nature has selected this novel function of CIITA to increase the fitness of human species. Its usage as a tool to induce expression of MHC-II genes and molecule in tumor cells has opened new possibilities to increase recognition of tumor antigens, their characterization and the possibilities to envisage realistic strategies of anti-tumor vaccination. This in turn has challenged the dogma that professional APC such as dendritic cells and macrophages are the exclusive cells capable to prime Th cells in adaptive immunity.

## Conflicts of interest

The authors declare that the research was conducted in the absence of any commercial or financial relationships that could be construed as a potential conflict of interest.
